# The Curve Visible on the Campbell-Robson Chart Is Not the Contrast Sensitivity Function

**DOI:** 10.3389/fnins.2021.626466

**Published:** 2021-03-09

**Authors:** Jessica Tardif, Marcus R. Watson, Deborah Giaschi, Frédéric Gosselin

**Affiliations:** ^1^Département de Psychologie, Université de Montréal, Montréal, QC, Canada; ^2^Department of Biology, York University, Toronto, ON, Canada; ^3^Department of Ophthalmology and Visual Sciences, The University of British Columbia, Vancouver, BC, Canada

**Keywords:** contrast sensitivity function, Campbell-Robson chart, spatial vision, low-level vision, psychophysics

## Abstract

The Campbell-Robson chart is a highly popular figure used in psychophysics and visual perception textbooks to illustrate the Contrast Sensitivity Function (CSF). The chart depicts a grating which varies logarithmically in spatial frequency (SF) from left to right and in contrast from bottom to top. Campbell and Robson’s (1964) intuition was that the boundary between the grating and the homogeneous gray area (below threshold) would trace the shape of the observer’s own CSF. In this paper, we tested this intuition. A total of 170 participants (96 adults and 74 children) adjusted the four parameters of a truncated log-parabola directly onto a Campbell-Robson chart rendition and completed a gold-standard CSF evaluation. We hoped that this procedure which requires a mere three clicks on the computer mouse, would speed up the measurement of the CSF to under a minute. Unfortunately, the only parameter of the truncated log-parabola fitted to the gold-standard CSF data that could be predicted from the Campbell-Robson chart data was the peak sensitivity for the adult participants. We conclude that the curve visible on the Campbell-Robson chart cannot be used practically to measure the CSF.

## Introduction

The human visual system analyses the complex luminance modulations that make up the visual stimulus with discrete channels, each tuned to a specific spatial frequency (SF) range that can be expressed in cycles per degree of visual angle (*cpd*; see De Valois and De Valois, 1990, for a review). Low SFs convey coarse information, such as the boundary between the sand and water on a beach, while high SFs can represent fine-grained information, such as the pole of a parasol several meters away (see Morrison and Schyns, 2001, and Ruiz-Soler and Beltran, 2006, for reviews). The threshold luminance contrast – the contrast required to detect simple sine wave gratings at a given level of performance or to discriminate their orientation – varies with SF. The contrast sensitivity function (CSF) depicts contrast sensitivity – the threshold’s reciprocal – as a function of SF. It has the shape roughly of an upside-down U (Campbell and Robson, 1964).

A wide variety of researchers measure the CSF today: it is a useful tool for screening and assessing spatial vision in many visual and cognitive impairments (e.g., Regan, 1991). The gold standard for measuring the CSF is to measure the contrast threshold for detecting the presence or for discriminating the orientation of 5–10 different SFs using the method of constant stimuli or a staircase method such as QUEST (for a review of different staircase methods, see Leek, 2001). When the method of constant stimuli is used to estimate the thresholds, each combination of SF and contrast is repeated 20 times or more, usually in random order. This procedure thus requires a minimum of 500 trials to evaluate five points on the CSF. When QUEST is used to measure the thresholds, each threshold estimate requires 40 trials or more. This method thus requires a minimum of 200 trials to evaluate five points on the CSF. In this article, we used QUEST for our gold-standard evaluation of the CSF. Several quicker and cruder alternatives have been proposed over the years (summarized below).

### Why Is It Important to Measure the CSF Quickly?

Contrast sensitivity is often measured in research settings, as an experimental variable, a covariable or to control for subjects’ visual health. There are various instances in which measuring the CSF with the gold-standard methods described above might be more difficult because of their duration. One such instance is when the population studied has difficulties to stay focused on a repetitive task for a long period. These populations include young children, older adults, and some persons who have neurological or psychological disorders lowering their attentional capacities, for example Attention Deficit Disorder or Major Depression. Another instance is simply when the other tasks participants complete already take a lot of time and completing a CSF assessment might add too much time. Similarly, if the CSF is used as a screening evaluation for excluding participants with atypical vision, a long evaluation might make this screening impractical.

In screening and regular vision evaluations, visual acuity is typically measured; it is the most useful tool to detect visual conditions, such as myopia. When contrast sensitivity is included in these regular test batteries, it usually consists of a measurement of perception threshold at a single SF (Pelli and Bex, 2013). Because the high SF end of the CSF drops to a null contrast sensitivity (i.e., no perception at 100% contrast) at a SF that approximates visual acuity, it can be estimated by a high-contrast Snellen-type chart. Similarly, the maximum contrast sensitivity of the CSF can be estimated, for example, by the Pelli-Robson chart described below (Pelli et al., 1988). Notwithstanding, these two types of screening tools each evaluate only one point on the CSF.

In some cases, visual acuity remains normal and contrast sensitivity is impaired (e.g., treated amblyopia: Huang et al., 2007; corrected severe myopia: Liou and Chiu, 2001; multiple sclerosis: Regan and Neima, 1983), it is therefore crucial to measure contrast sensitivity to detect these impairments. Further, in many cases, contrast sensitivity is only affected for a subset of SFs. In these cases, the isolated deficit cannot be observed if the method used does not encompass the SFs for which sensitivity is altered, which means that it is not enough to use the Snellen as well as the Pelli-Robson charts. In these cases, measuring the entire CSF is required to screen for visual impairments. For example, these cases include disorders that primarily affect sensitivity to high SF: amblyopia (in all studies 12 cpd and above; Hess and Howell, 1977; Bradley and Freeman, 1981; Sjöstrand, 1981; Howell et al., 1983; Levi et al., 1994; however, Barollo et al., 2017 observed low contrast sensitivity in amblyopia at all SFs), macular degeneration (in all studies 6 cpd and above; Wolkstein et al., 1980; Marmor, 1986; Kleiner et al., 1988; Stangos et al., 1995; Sunness et al., 1997) and high myopia (Thorn et al., 1986; Collins and Carney, 1990; Liou and Chiu, 2001). The SF ranges at which sensitivity is impaired seem to be case-dependent for other disorders. For example, multiple sclerosis usually affects middle SFs, but it also affects high and low SFs in some patients (Nordmann et al., 1987; Ashworth et al., 1989). Cataracts most often affect sensitivity only for intermediate and high SFs – about 2 cpd and above –, while in other cases they also affect lower SFs (Hess and Woo, 1978; Elliott et al., 1989; Elliott and Hurst, 1990; Pardhan and Gilchrist, 1991; Lewis et al., 1992; Superstein et al., 1997; Shandiz et al., 2011).

### How Can the CSF Evaluations Be Shortened?

Over the years, CSF evaluation methods have been shortened in many clever ways. Before 1990, a few paper-based charts were introduced as quick and cheap methods for estimating the CSF. The Arden plates (1978), for example, were simple gratings of specific SFs printed on cards, with contrast varying from top to bottom. These cards were at first completely covered by a second opaque gray card which was moved slowly to reveal increasing contrast levels. Subjects verbally reported when they perceived the grating, and completed the procedure with different SFs, allowing the experimenter to trace a threshold curve. The results, although reported as unreliable (Robson, 1993), remain useful as a screening tool (Woods et al., 1998).

The Vistech chart (Ginsburg, 1984) is a cardboard chart featuring a grid of Gabor patches of different SFs and three orientations. Subjects simply report the orientation of the gratings until the contrast is too low for them to perceive it. An advantage of this method is that the task is objective, unlike in the case of the Arden plates. Subjects complete the Vistech chart in about 6 min (Robson, 1993). Threshold estimates tend to be noisy, however, because each SF and contrast combination is only presented once (Robson, 1993). While the Vistech thresholds correlate well with a method which includes a larger number of trials (Leat and Woo, 1997), its reliability is low (Rubin, 1988; Reeves et al., 1991). Furthermore, cardboard charts fade with time, altering the contrast of the printed gabors or letters.

More recently, computerized tests were combined with adaptive algorithms to measure the CSF. The Freiburg Visual Acuity Test (FrACT; Bach, 1996, 2006) is one such example. FrACT contains different tasks to evaluate acuity and contrast sensitivity. In one of these, the participant is asked to identify the orientation of a sinusoidal grating of a specified spatial frequency among four possibilities (horizontal, vertical, and the obliques). The contrast threshold of this stimulus is measured efficiently using the Best-PEST adaptive algorithm (Lieberman and Pentland, 1982). The CSF of the participant can be estimated by running this task multiple times with gratings of different spatial frequencies. A software implementing FrACT is freely available^1^.

In Quick CSF (Lesmes et al., 2010), another example of an adaptive method and the fastest to date, each stimulus is chosen in light of the participant’s past accuracy to present the stimulus that has the greatest potential for new information. This method is highly efficient, taking only 10 min for an accurate measurement and 2 min for a broad evaluation (Lesmes et al., 2010). It has been validated for achromatic contrast sensitivity (Dorr et al., 2013), as well as for chromatic contrast sensitivity (Kim et al., 2017).

The CSF can also be measured quickly using electroencephalography (EEG). The fastest method – the sweep VEP (e.g., Seiple et al., 1984; Allen et al., 1986; Norcia et al., 1989) – uses gratings contrast-reversing at 12 Hz, for example, and increasing either in SF or contrast over 10 s. Participants view these stimuli while their electrophysiological scalp response is recorded. A discrete Fourier transform is applied to the signal to measure the amplitude of the electrophysiological response at the frequency of the contrast-reversals. The amplitude associated with a particular SF is related to the contrast threshold at this SF. The measure takes only about 10 s per sweep, in addition to the time needed to install the electrodes – as little as two occipital electrodes and a reference. However, it requires EEG equipment as well as knowledge of how to use it, which are not readily accessible to everybody who might want to measure the CSF.

### The Campbell-Robson Chart

The Campbell-Robson chart (Campbell and Robson, 1964; Ratliff, 1965) is a highly popular figure used in several psychophysics and visual perception textbooks to illustrate the CSF (e.g., Lu and Dosher, 2014; Wolfe et al., 2018). The chart depicts a grating which varies logarithmically in spatial frequency (SF) from left to right and in contrast from bottom to top. Campbell and Robson’s (1964) intuition was that the boundary between the grating and the homogeneous gray area (below threshold) would trace the shape of the observer’s own CSF. Surprisingly, it appears that no one has tested if the Campbell-Robson chart can indeed be used to measure the CSF.

Here, we asked adults and children with normal or corrected-to-normal vision to adjust directly the *truncated log-parabola* to the curve visible on a rendition of the Campbell-Robson chart. The truncated log-parabola was chosen here because of its simplicity and its good fit to the CSF curves of observers with normal vision (e.g., Lesmes et al., 2010). Participants adjusted the four parameters of this curve in only three mouse clicks. Then, we attempted to predict the parameters of the truncated log-parabola adjusted on the contrast thresholds of seven SFs estimated using the QUEST algorithm from those of the truncated log-parabola adjusted directly on the Campbell-Robson chart in adults (*N* = 100) and in children (*N* = 81). If successful, this procedure, which requires a mere three clicks on the computer mouse, would speed up the measurement of the CSF to under a minute.

## Materials and Methods

### Participants

A total of 103 adults were recruited at Université de Montréal (*N* = 83) or at The University of British Columbia (*N* = 20). One participant was excluded due to technical problems during data collection, and data for six additional outlier participants were removed (see section “Results”). The final adult sample comprised 96 individuals between 18 and 35 years of age [median of 22.0 (interquartile range: 3); 34 men]. Ninety-nine children were recruited at The University of British Columbia. An incomplete dataset was obtained for 15 children due to various reasons: technical problems (*N* = 1), the child not feeling well (*N* = 1), or general restlessness, distractedness, not following instructions or lack of time (*N* = 13). Eight outlier children’s data were removed and two additional children’s data were removed because the truncated log-parabola did not properly adjust to the thresholds measured with the gold-standard method (see section “Results”). The final sample comprised 74 children between 4.9 and 17.7 years old [median of 11.1 (interquartile range: 4.9)]. All participants were neurotypical and received a small monetary compensation for their participation. Participants recruited at Université de Montréal (all adults) reported having normal or corrected-to-normal vision, and participants recruited at The University of British Columbia (all children and some adults) completed the Regan chart to determine monocular distance visual acuity. The study was conducted in accordance with the Code of Ethics of the World Medical Association (Declaration of Helsinki), and approved by the Children’s and Women’s Research Ethics Board at the University of British Columbia as well as the Comité d’éthique de la recherche en éducation et en psychologie (CEREP) at Université de Montréal. Informed consent was obtained from each adult participant, or parent/guardian as well as verbal or written assent from each child participant.

### Apparatus

The experimental programs were run on Macintosh computers in the Matlab (MathWorks Inc.) environment, using functions from the Psychophysics Toolbox (Brainard, 1997; Pelli, 1997; Kleiner et al., 2007). Participants were seated in a dim-lighted room. For participants recruited at Université de Montréal (only adults), all stimuli were presented on 27-inch Asus VG278H monitors (1920 × 1080 pixels at 120 Hz), calibrated to allow linear manipulation of luminance. Luminance ranged from 0.33 to 245 cd/m^2^ (measured with a Samsung SyncMaster 753df photometer). A chinrest was used to maintain a constant viewing distance. For participants recruited at The University of British Columbia (all children and some adults), stimuli were presented on a 24-inch A1267 Apple Cinema Display (1920 × 1200 pixels at 60 Hz); luminance ranged from 1.6 to 159 cd/m^2^ (measured with a Minolta LS-110 photometer).

### Procedure

#### Three-Click CSF Method

**FIGURE 1:**
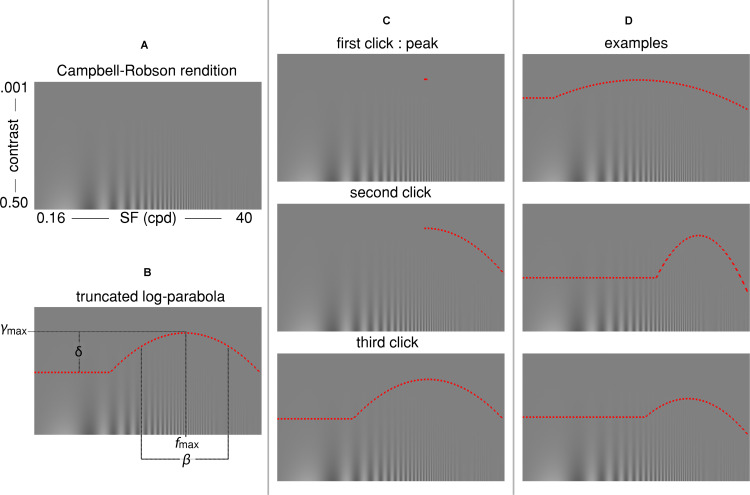
**(A)** The Campbell-Robson chart. **(B)** The truncated log-parabola is defined by four parameters: f_max_ and y_max_ are the peak of the curve, respectively, the frequency at which it peaks and the sensitivity at this point; β is the Full-Width at Half-Maximum (FWHM); and δ is the truncation parameter in the lower spatial frequency range. **(C)** Three computer mouse clicks are required for adjusting the curve: The first click determines f_max_ and y_max_, the coordinates of the peak. The second click determines β, the sensitivity for high spatial frequencies. The last click determines the truncation parameter δ, or the sensitivity to low spatial frequencies. **(D)** Three examples of the shape individual curves could take, depending on parameters.

Each participant completed three runs of the three-click CSF. On each run, a Campbell-Robson chart was generated to cover the whole computer screen (Figure 1A). SFs varied logarithmically from 0.16 to 40 cycles per degree in 14.4° of visual angle (from left to right) and Michelson contrast varied logarithmically from 0.001 to 0.5 (from bottom to top) in 8.12° of visual angle for the Université de Montréal participants and 9° of visual angle for The University of British Columbia participants. We used the noisy-bit method which uses spatial pooling to increase luminance resolution beyond 8 bits (Allard and Faubert, 2008). Participants were instructed to adjust the truncated log-parabola directly onto the rendition of the Campbell-Robson chart (see Figure 1D for examples of the truncated log-parabola with differing parameters). Adults used a computer mouse to adjust the curve’s parameters, children used either a computer mouse or a game controller.

The truncated log-parabola is defined as

$$\text{f}\,\left(x\right)=\left\{ \begin{array}{c} \log_{10}\left(y_{\max}\right)-\delta ,\\ \text{if}\,x<f_{\max}\,\text{and}\,\text{f}\,\left(x\right)<\log_{10}\left(y_{\max}\right)-\delta \\ \log_{10}\left(y_{\max}\right)-{\left(\frac{\log_{10}x-\log_{10}f_{\max}}{\beta \cdot \log_{10}\left(2\right)/2}\right)}^{2},\\ \text{otherwise} \end{array}\right.$$

Equation 1 – truncated log-parabola

where f(*x*) is the contrast sensitivity threshold, *x* is the spatial frequency, *y*_max_ is the peak sensitivity, *f*_max_ is the SF of this peak sensitivity, *β* is the Full-Width-at-Half-Maximum (FWHM) of the log-parabola in octaves, and *δ* is the truncation parameter. The curve was specified by the latter four quantities which were determined by three computer mouse clicks (see Figure 1C). First, participants were instructed to place a small horizontal line segment at the highest point at which they could see “stripes” on the displayed Campbell-Robson chart. This first click served to establish the peak contrast sensitivity (*y*_max_) and the SF at which it peaked (*f*_max_). Next, the right half of a log-parabola peaking at (*f*_max_, *y*_max_) appeared as a dotted red curve on the displayed Campbell-Robson chart. Participants were instructed to adjust the width of this curve so that the “stripes” were visible underneath it but invisible over it by moving the computer mouse along the *x*-axis, and to click on the mouse button when they were satisfied with the adjustment. This second click defined the FWHM of the log-parabola (*β*), or the contrast sensitivity for the mid to high SF. Finally, a complete truncated parabola peaking at (*f*_max_, *y*_max_) and with a FWHM equal to *β* was overlaid on the Campbell-Robson chart as a dotted red curve. Participants were instructed to adjust the height of the truncated portion of this curve so that the “stripes” were visible underneath it but invisible over it by moving the computer mouse along the *y*-axis, and to click on the computer mouse button when they were satisfied with the adjustment. This third and last click determined the truncation parameter (*δ*) of the truncated log-parabola, or the contrast sensitivity for low SFs. These instructions were given to adults while they performed their first of three three-click CSF runs. Children were given adapted instructions in the form of an animated PowerPoint story prior to their three three-click CSF runs. Participants were not given any instruction about fixation and could freely explore the stimulus.

#### Gold-Standard CSF Method

We compared the four truncated log-parabola parameters adjusted on the Campbell-Robson chart with the four parameters of the truncated log-parabola fitted to the contrast sensitivity thresholds measured for sinusoidal gratings of seven different SFs using the QUEST staircase method (Watson and Pelli, 1983). This psychophysical adaptive method has often been used to measure the CSF (e.g., Burr et al., 1994; Carrasco et al., 2000).

All participants completed 336 trials (48 trials for each SF), divided into four blocks of 84 trials. Michelson contrast thresholds were measured independently for each of seven SFs: 0.5, 0.99, 1.96, 3.87, 7.66, 15.16, and 30 cycles per degree. Gratings were revealed through a Gaussian window with a FWHM equal to 2° of visual angle. Noisy-bit dithering was applied to every stimulus (Allard and Faubert, 2008). The starting values of the contrast threshold estimates were determined using the average of the Gabor data for all subjects from ModelFest (Carney et al., 2000). The order of presentation of the different SFs was randomized. The gratings were presented equiprobably horizontally or vertically on each trial. Participants were instructed to indicate the orientation of the gratings using the arrows on the computer keyboard. As with the three-click CSF, children were given adapted instructions and could answer using the buttons of a game controller. Contrast was adjusted using the QUEST algorithm for each SF, independently, to reach a correct rate of 82%.

## Results

### Gold-Standard CSF Evaluation

Threshold contrast levels were obtained from the final QUEST contrast threshold estimates for every tested SF. To compare the QUEST to the Campbell-Robson chart adjustment, we fitted a truncated log-parabola (Equation 1 and illustrated in Figure 1) to the sensitivity levels measured by QUEST for each participant. Apart from a few exceptions, the function fitted very well to these data [Adults: median *R*^2^ = 0.96 (interquartile range: 0.04); Children: median *R*^2^ = 0.93 (interquartile range: 0.07)]. We excluded two children from further analysis because the R^2^ of their fit was lower than 0.5. We also excluded two adults and five children because at least one of their fitted parameters was an outlier (*Z* > 3.00).

**FIGURE 2:**
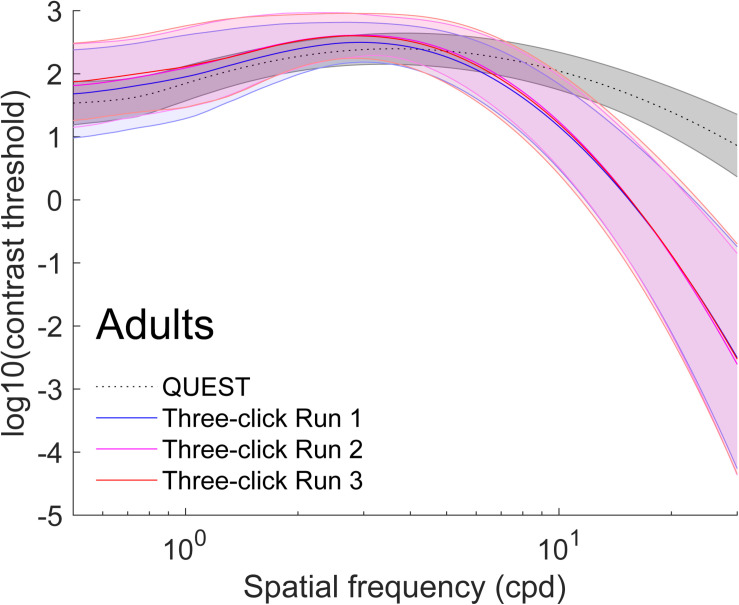
Average (±1 standard deviation in shaded area) contrast sensitivity functions for adults (*N* = 96). To obtain these curves, we first computed four truncated log-parabola curves for each individual: one adjusted on their sensitivity measured using QUEST, and three for each run adjusted onto the Campbell-Robson. Then, each curve was averaged across participants point-by-point. The average as measured using QUEST to find thresholds is presented in dotted gray (±1 standard deviation in shaded area) and the average curve adjustments on the Campbell-Robson chart are presented in blue (run 1), pink (run 2), and red (run 3).

**FIGURE 3:**
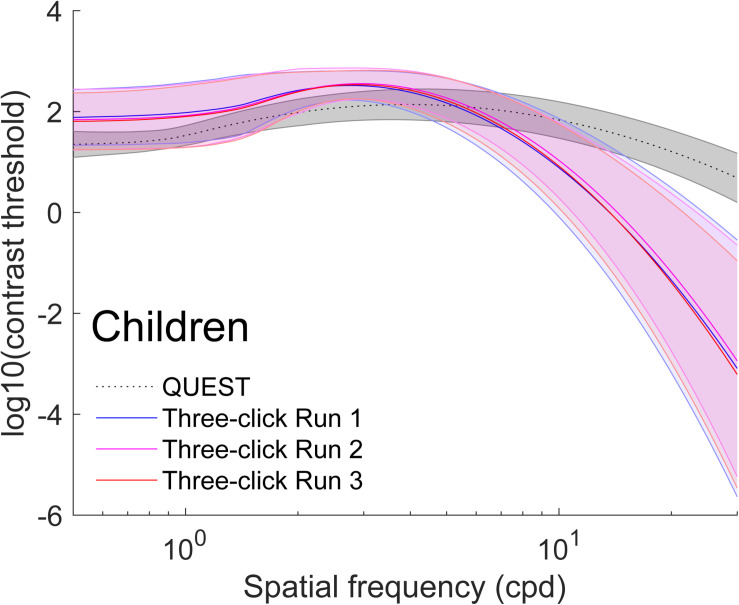
Average (±1 standard deviation in shaded area) contrast sensitivity functions for children (*N* = 74). To obtain these curves, we first computed four truncated log-parabola curve for each individual: one adjusted on their sensitivity measured using QUEST, and three for each run adjusted onto the Campbell-Robson. Then, each curve was averaged across participants point-by-point. The average, as measured using QUEST to find thresholds, is presented in dotted gray (±1 standard deviation in shaded area), and the average curve adjustments on the Campbell-Robson chart are presented in blue (run 1), pink (run 2), and red (run 3).

The average of all curves measured with this method are presented in gray in Figure 2 (adult data) and Figure 3 (child data). To obtain these figures, we adjusted a truncated log-parabola on the sensitivity levels obtained by each participant. Then, we averaged all the curves point-by-point.

### Three-Click CSF Method Evaluation

We excluded four adults and three children from further analysis because at least one of their four three-click CSF parameters were outliers (*Z* > 3.00). Average parameters of the truncated log-parabola adjusted on the Campbell-Robson chart for each of the three runs are presented in Tables 1 and 2. The point-by-point average of all curves measured with the three-click CSF method are plotted for each run, in red, pink and blue on Figure 2 (adult data) and Figure 3 (child data).

**TABLE 1:** Average parameters (*ß*, δ, *f*_max_, and *y*_max_) defining the truncated log-parabola for adult participants (*N* = 96) when the contrast sensitivity function was measured using the Campbell-Robson chart (Runs 1, 2, and 3 independently), and when it was measured using QUEST to find perceptive thresholds (Q).

**Task**	**Run**	***ß***	**δ**	***f*_max_**	***y*_max_**
Campbell-Robson	1	3.24 (SD = 0.96)	1.08 (SD = 0.76)	2.96 (SD = 1.23)	550.04 (SD = 364.52)
	2	3.15 (SD = 0.91)	1.06 (SD = 0.75)	3.03 (SD = 1.33)	729.50 (SD = 461.71)
	3	3.19 (SD = 0.85)	1.01 (SD = 0.70)	2.96 (SD = 1.32)	722.51 (SD = 504.89)
QUEST		5.13 (SD = 1.02)	0.90 (SD = 0.36)	3.62 (SD = 0.82)	294.33 (SD = 135.19)

**TABLE 2:** Average parameters (*ß*, δ, *f*_max_, and *y*_max_) defining the truncated log-parabola for child participants (*N* = 74) when the contrast sensitivity function was measured using the Campbell-Robson chart (Runs 1, 2, and 3 independently), and when it was measured using QUEST to find perceptive thresholds (Q).

**Task**	**Run**	***ß***	**δ**	***f*_max_**	***y*_max_**
Campbell-Robson	1	3.23 (SD = 0.97)	0.83 (SD = 0.49)	2.68 (SD = 0.89)	537.55 (SD = 371.20)
	2	3.17 (SD = 0.89)	0.88 (SD = 0.54)	2.82 (SD = 0.88)	554.14 (SD = 333.73)
	3	3.09 (SD = 0.83)	0.86 (SD = 0.43)	2.81 (SD = 0.89)	527.71 (SD = 295.59)
QUEST		5.01 (SD = 0.75)	0.84 (SD = 0.24)	3.94 (SD = 0.85)	168.18 (SD = 86.48)

On average, the Campbell-Robson chart curve adjustment overestimated low-to-mid SF sensitivity and underestimated high SF sensitivity. Specifically, as observed in Figure 2, mean peak contrast sensitivity was higher for the Campbell-Robson than the gold-standard measure, as determined using a paired *t* test [*y_*max*_;* Adults:*t*(95) = 10.45*; p <* 0.0001*;* Children: *t*(73) = 10.82*; p <* 0.0001]. While the difference between the peaks of the two methods seems bigger for children than for adults, the method × age group interaction was not significant [*F*(1,168) = 0.37; *p* = 0.55]. At higher SF, mean SF at which contrast sensitivity peaked [*f*_max;_ *t*(95) = −4.59; *p* < 0.001; Children: *t*(73) = −9.66; *p* < 0.0001] and mean log-parabola width [*β*; Adults: *t*(95) = −17.25; *p* < 0.0001; Children: *t*(73) = −17.35; *p* < 0.0001] were lower for the three-click CSF than the gold-standard measure. At low SF, the parameter *δ* statistically differed for adults but not for children [*δ;* Adults: *t*(95) = 2.31; *p* = 0.02; Children: *t*(73) = 0.32; *p* = 0.75], but since it measures the difference in sensitivity between the peak and sensitivity at low SF, it is more difficult to interpret. Instead, we compared sensitivity at the lowest spatial frequency (0.5 cpd). At that specific point, it is higher when measured with the three-click method [Adults: *t*(95) = 3.60; *p* < 0.001; Children: *t*(73) = 7.76; *p* < 0.0001].

### Extracting Information From the Campbell-Robson Chart

The main objective of this paper was to verify if it is possible to extract CSF information directly from adjusting a specific curve to the Campbell-Robson chart. To investigate this question, we adopted a data-driven approach. More specifically, we used machine learning methods to verify if we can predict the CSF using only the parameters adjusted on the Campbell-Robson chart.

We trained models to predict the four parameters evaluated from the individual gold-standard thresholds measured with QUEST using the four individual parameters adjusted directly on the Campbell-Robson chart with the three-click CSF method. We trained different sets of models for children and for adults and evaluated their performance using 6-fold cross-validation. Specifically, we did 100 iterations of the following. First, the dataset was randomly split into a test set of 16 random observations (12 for children) and a training set. Then, the training set was randomly split evenly into five sets of the same size as the test set. Each of these five sets in turn was set aside and SVMs with Gaussian kernel whose FWHM varied between 0.1 and 5 in increments of 0.1 were trained on the remaining 4/5 sets. Their Root-Mean-Square Errors (RMSE) were evaluated on the 1/5 set that was put aside. The best model (lowest RMSE) was chosen across the five folds and applied to calculate a RMSE and a R^2^ on the independent test set. To verify how much information we gain by adjusting the curve on the Campbell-Robson chart more than once, this 100-iteration procedure was repeated three times: (1) using the parameters of the first run, (2) using the parameters of the first two runs, and (3) using the parameters of the three runs.

To compare the RMSE we obtained with the null hypothesis, we also ran a permutation test. The exact same steps were followed in the permutation test, but on each iteration the dataset was randomly permuted so that independent variables (parameters adjusted on the Campbell-Robson chart) were matched with dependent (parameters measured with QUEST) variables of a different, random participant. In other words, we ran the same analysis but with data for which the independent and dependent variables were not linked.

**FIGURE 4:**
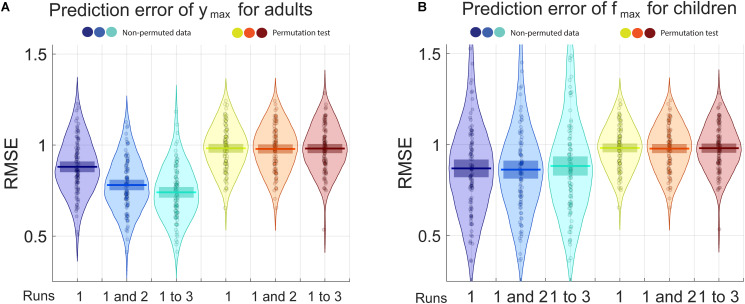
The blue pirate plots show the Root Mean Square Error (RMSE) of the prediction of *y*_*max*_, the truncated log-parabola’s maximal height, for adults, and of fmax, the spatial frequency at the curve’s maximal height, for children. The darkest, middle and lightest blue pirate plots show the RMSE of the predictions from models based, respectively, on the first, on the first and the second, and on all three three-click CSF runs. The pirate plots with warm colors show the null hypothesis obtained using a permutation test (see also Supplementary Figures S1, S2).

**FIGURE 5:**
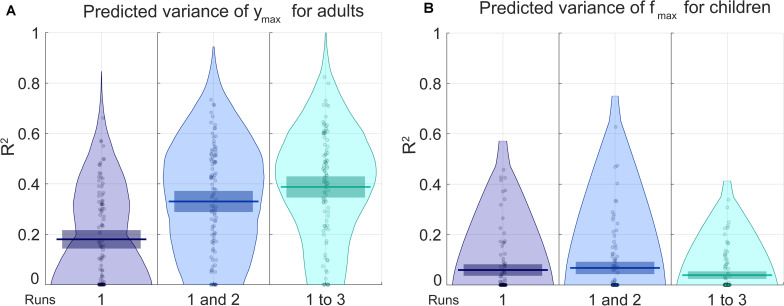
The blue pirate plots show the explained variance (R^2^) of the predicted of *y*_*max*_, which defines the truncated log-parabola’s maximal height, for adults, and of *f*_*max*_, the spatial frequency at the curve’s maximal height, for children. The darkest, middle and lightest blue pirate plots show the R^2^ of the predictions from models based, respectively, on the first, on the first and the second, and on all three three-click CSF runs. The pirate plots with warm colors show the null hypothesis obtained using a permutation test (see also Supplementary Figures S3, S4).

For adults, the only parameter for which the prediction was better with the non-permuted data than the permuted data is *y*_*max*_, the maximal sensitivity (highest Y point). For children, that parameter is *f*_*max*_ (the frequency at which the maximal sensitivity is attained). RMSE and R^2^ are presented, respectively, in Figures 4, 5 for the prediction of (Figure 4A) *y*_*max*_ for adults and (Figure 4B) *f*_*max*_ for children. Progressively adding data from more than one run of fitting the curve on the Campbell-Robson chart (i.e., twice or three times) lowers the error for predicting *y*_*max*_ for adults, as can be observed in Figure 4A. For predicting *y*_*max*_ for adults, RMSE is an average of 0.88 using one run of data, and lowers to 0.78 using two runs of data, and 0.74 using three runs of data. We can predict 38.8% of the variance using three runs of data. Using the best model from the 100 iteration, we predict 45.7% of the variance of *y*_*max*_. For the other three parameters measured with adults, the RMSE after three runs was of 0.88, 0.86, and 0.92, respectively, for beta, delta, and *f*_*max*_, equivalent to predicting 2.3, 14.6, and 6.2% of the variance of new data.

For predicting *f*_*max*_ for children, RMSE is of an average of 0.87 using one run of data, and does not lower when adding successive runs of data. Average RMSE using three runs of data is of 0.88. This does not translate to being able to predict much of the variance for *f*_*max*_, the best R^2^ is of 0.068 using two runs of data. In Supplementary Material, RMSE and *R*^2^ values are presented in the form of graphs for each iteration, each parameter and each number of runs included.

## Discussion

In this article, we examined the mainstream belief, originating from Campbell and Robson’s (1964) seminal work, that the boundary between the grating and the homogeneous gray area in the Campbell-Robson chart traces the shape of an observer’s own CSF. Participants adjusted the truncated log-parabola directly to the curve visible on the Campbell-Robson chart. We call this procedure the “three-click CSF method.” They also completed a gold-standard evaluation of their CSF, using QUEST to adjust the contrast of sinusoidal gratings of different spatial frequencies on a trial-to-trial basis. We found that, on average, the three-click CSF method overestimated low-to-mid SF sensitivity and underestimated the high SF sensitivity compared to our gold-standard method for measuring the CSF. We then trained support vector machine models with radial-basis function kernels to predict the gold-standard CSF from the four parameters obtained with the three-click method. Our goal was to extract as much CSF information as possible – linear and non-linear – from the Campbell-Robson chart. We found that, in adults, 42% of the variance of the maximal sensitivity (the *y*_*max*_ parameter of the truncated log-parabola) could be predicted using the best support vector machine model. None of the other parameters could be predicted in adults, nor any parameter whatsoever in children.

Dorr et al. (2017) asked their participants to tap on the peak of the curve visible on a Campbell-Robson chart presented on a tablet – the coordinates of this peak should match closely our *f*_*max*_ and *y*_*max*_. They found a linear relationship between these coordinates and the area under the curve obtained using the Quick CSF method (*r* = 0.63 for x coordinate or *f*_*max*_ and *r* = 0.58 for y coordinate or *y*_*max*_). The area under the curve measure can be understood as a global contrast sensitivity measure. For adults, we also found significant correlations between the area under the curve calculated from our gold-standard data and, first, *f*_*max*_ measured with the three-click method in all three runs (*r* = 0.20, *p* = 0.047; *r* = 0.32, *p* < 0.001; *r* = 0.23, *p* = 0.03, respectively, for the first, second, and third run) and, second, *y*_*max*_ measured with the three-click method in the second and third runs (*r* = 0.17, *n.s.*; *r* = 28, *p* = 0.01; *r* = 0.30, *p* = 0.003, respectively, for the first, second, and third run). These correlations, however, were not significant for children.

The discrepancies observed between the three-click CSF and the gold-standard results could be due to one or a combination of the many differences between the methods. For example, while our gold-standard CSF method is objective, the three-click CSF method is subjective. Thus, in the former, QUEST searched for the thresholds associated with a correct rate of 82% for each spatial frequency and participant. In the latter, the correct rate is undefined. The two tasks also differ drastically in stimulus size: 14.42° of visual angle for the Campbell-Robson chart used for the three-click CSF method vs. about 2° of visual angle for the sinusoidal gratings used for the gold-standard method. The three-click CSF method thus required several saccades to foveate the parts of the Campbell-Robson chart relevant to the different adjustment clicks. These eye movements could also have led to confounding grating aftereffects – resulting from the fixation of a contrasted region of the Campbell-Robson chart – for actual gratings in the “gray” regions of the chart. Moreover, each SF occupies a much smaller area in the Campbell-Robson chart (1 pixel or about 0.63 min of arc) than in the gold-standard method (2° of visual angle). This may also have had an effect on the observed sensitivity, especially for the high SF where the represented SFs change rapidly.

Is it possible that the truncated log-parabola, the curve that we chose to fit onto the Campbell-Robson chart, does not capture the CSF information on the Campbell-Robson chart? This curve fits very well to our gold-standard thresholds [Adults: median *R*^2^ = 0.96 (interquartile range: 0.04); Children: median *R*^2^ = 0.93 (interquartile range: 0.07)]. It also fits very well to the ModelFest thresholds [median *R^2^* = 0.99 (interquartile range: 0.01)]. In other words, it provides a very good approximation of the gold-standard CSF. In fact, this is one of the reasons it is at the core of the Quick CSF method (Lesmes et al., 2010). Could it be, however, that the truncated log-parabola doesn’t fit the curve visible on the Campbell-Robson chart very well but that, nonetheless, this curve contains useful information about the CSF? It is difficult to give objective evidence for or against this possibility. Our participants reported that the curve adjustment was easy, with the possible exception of the third, and last, click that measured the truncation parameter (*δ*) or the contrast sensitivity for low SFs. A few participants reported dips to which they could not adjust the curve. A more quantitative evidence of the adequacy of the curve is the intra-subject consistency. For all truncated log-parabola parameters and between all pairs of runs, the average Pearson reliability was 0.70 (SD = 0.10; it ranged from 0.54 for *δ* between childrens’ runs 1 and 2 and 0.88 for *y*_*max*_ between adults’ runs 2 and 3). Thus the truncated log-parabola appears to fit well the Campbell-Robson chart curve at least subjectively. This truncated log-parabola, however, is different from the one that fits the gold-standard CSF.

In sum, the short answer to our opening question is: The Campbell-Robson chart cannot be used to measure the CSF. It does predict contrast sensitivity (*y*_*max*_) but other rapid methods to measure contrast sensitivity are already available and widely used, such as the Pelli-Robson chart. The Campbell-Robson chart should remain a useful educational tool to teach students about the broad shape of the CSF.

## Data Availability Statement

The raw data supporting the conclusions of this article will be made available by the authors, without undue reservation.

## Ethics Statement

The studies involving human participants were reviewed and approved by the Comité d’éthique de la recherche en éducation et en psychologie de l’Université de Montréal. The patients/participants provided their written informed consent to participate in this study.

## Author Contributions

JT, MW, and FG programmed the experiments. JT recruited and tested the adult participants, drafted the manuscript, and edited by FG and DG. MW recruited and tested the child participants. JT and FG worked on data analysis and interpretation. All authors contributed to the development of the project. DG and FG supervised all aspects of the study.

## Conflict of Interest

The authors declare that the research was conducted in the absence of any commercial or financial relationships that could be construed as a potential conflict of interest.

## Footnotes

1 https://michaelbach.de/fract/download.html
