# Supplementary material for: The Curve Visible on the Campbell-Robson Chart Is Not the Contrast Sensitivity Function
Source: Front Neurosci. 2021 Mar 9;15:626466. doi: 10.3389/fnins.2021.626466 (PMC7985182; doi:10.3389/fnins.2021.626466)
Supplement: Supplementary file 1 [file Data_Sheet_1.pdf]

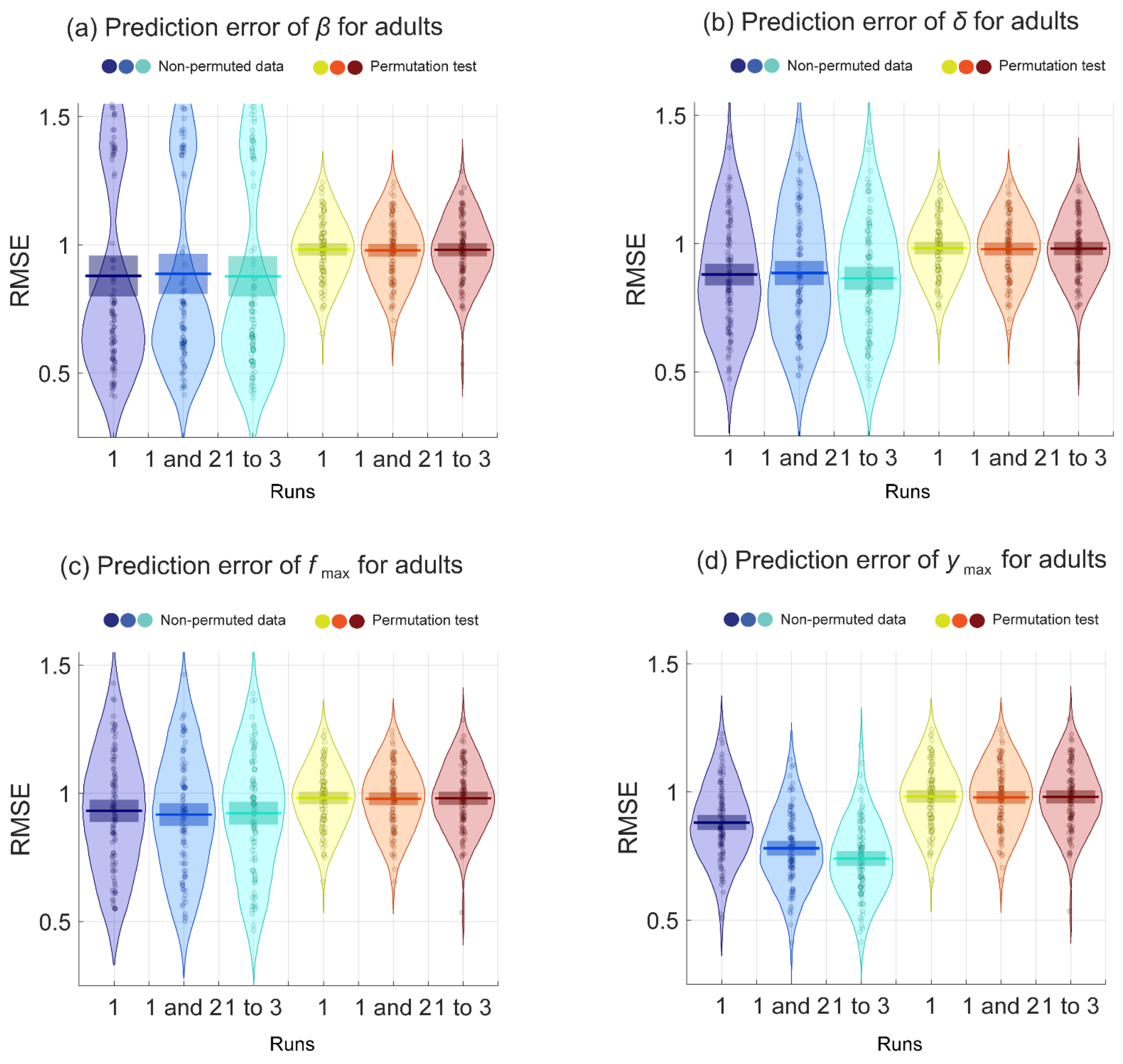

Figure S1 – The blue pirate plots show the Root Mean Square Error (RMSE) of the CSF parameter predictions for adult participants: in (a) of *β*, the truncated log-parabola's full-width-at-half-maximum; in (b) of *δ*, the curve's truncation parameter; in (c) of f_{max}, the spatial frequency at the curve's maximal height; and in (d) of y_{max}, the curve's maximal height. The darkest, middle and lightest blue pirate plots show the RMSE of the predictions from models based, respectively, on the first, on the first and the second, and on all three three-click CSF runs. The pirate plots with warm colors show the null hypothesis obtained using a permutation test.

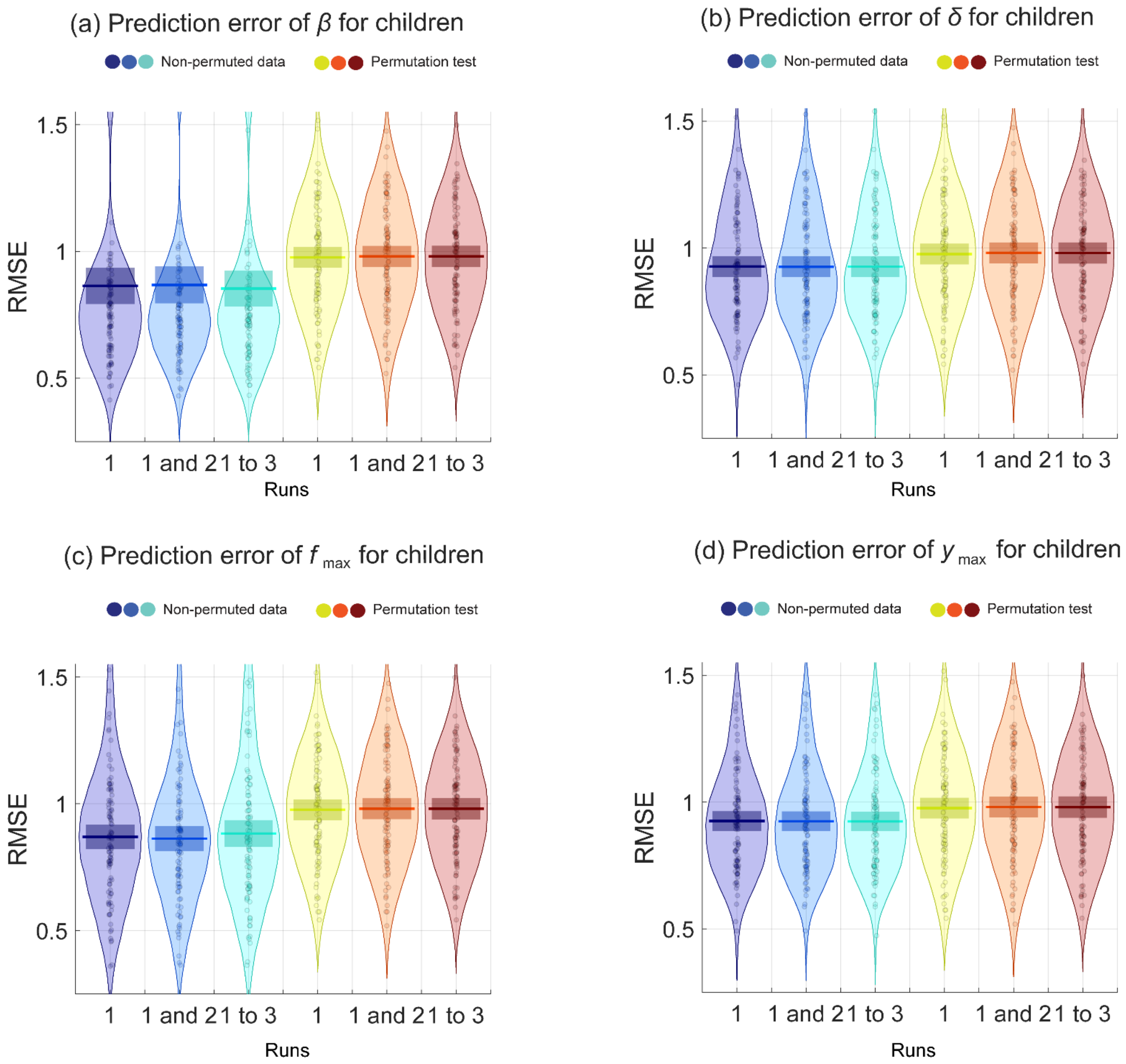

Figure S2 – The blue pirate plots show the Root Mean Square Error (RMSE) of the CSF parameter predictions for children participants: in (a) of β, the truncated log-parabola's full-width-at-half-maximum; in (b) of δ, the curve's truncation parameter; in (c) of f_{max}, the spatial frequency at the curve's maximal height; and in (d) of y_{max}, the curve's maximal height. The darkest, middle and lightest blue pirate plots show the RMSE of the predictions from models based, respectively, on the first, on the first and the second, and on all three three-click CSF runs. The pirate plots with warm colors show the null hypothesis obtained using a permutation test.

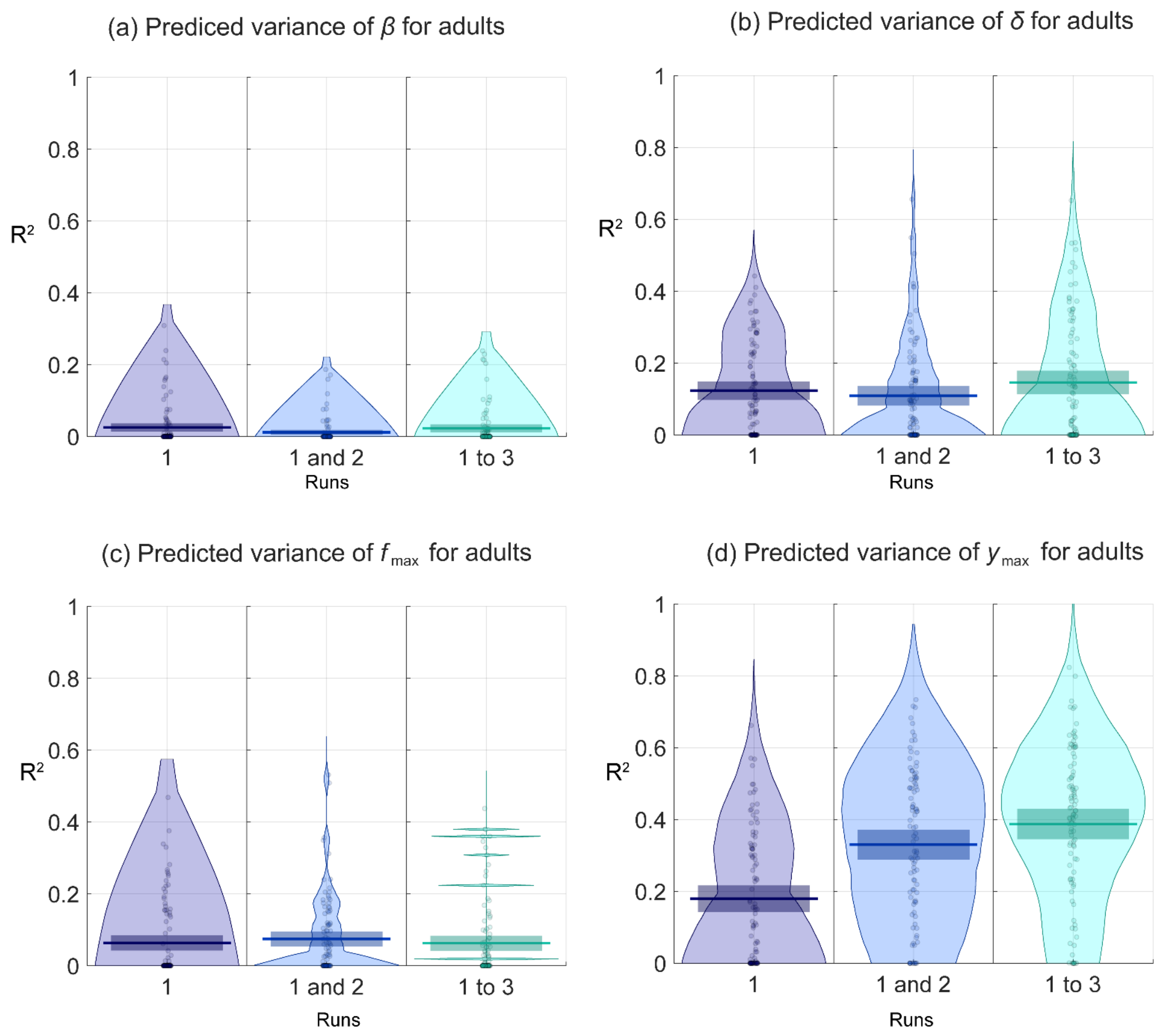

Figure S3 – The blue pirate plots show the explained variance (R^2) of the CSF parameter predictions for adult participants: in (a) of β, the truncated log-parabola's full-width-at-half-maximum; in (b) of δ, the curve's truncation parameter; in (c) of f_{max}, the spatial frequency at the curve's maximal height; and in (d) of y_{max}, the curve's maximal height. The darkest, middle and lightest blue pirate plots show the R^2 of the predictions from models based, respectively, on the first, on the first and the second, and on all three three-click CSF runs. The pirate plots with warm colors show the null hypothesis obtained using a permutation test.

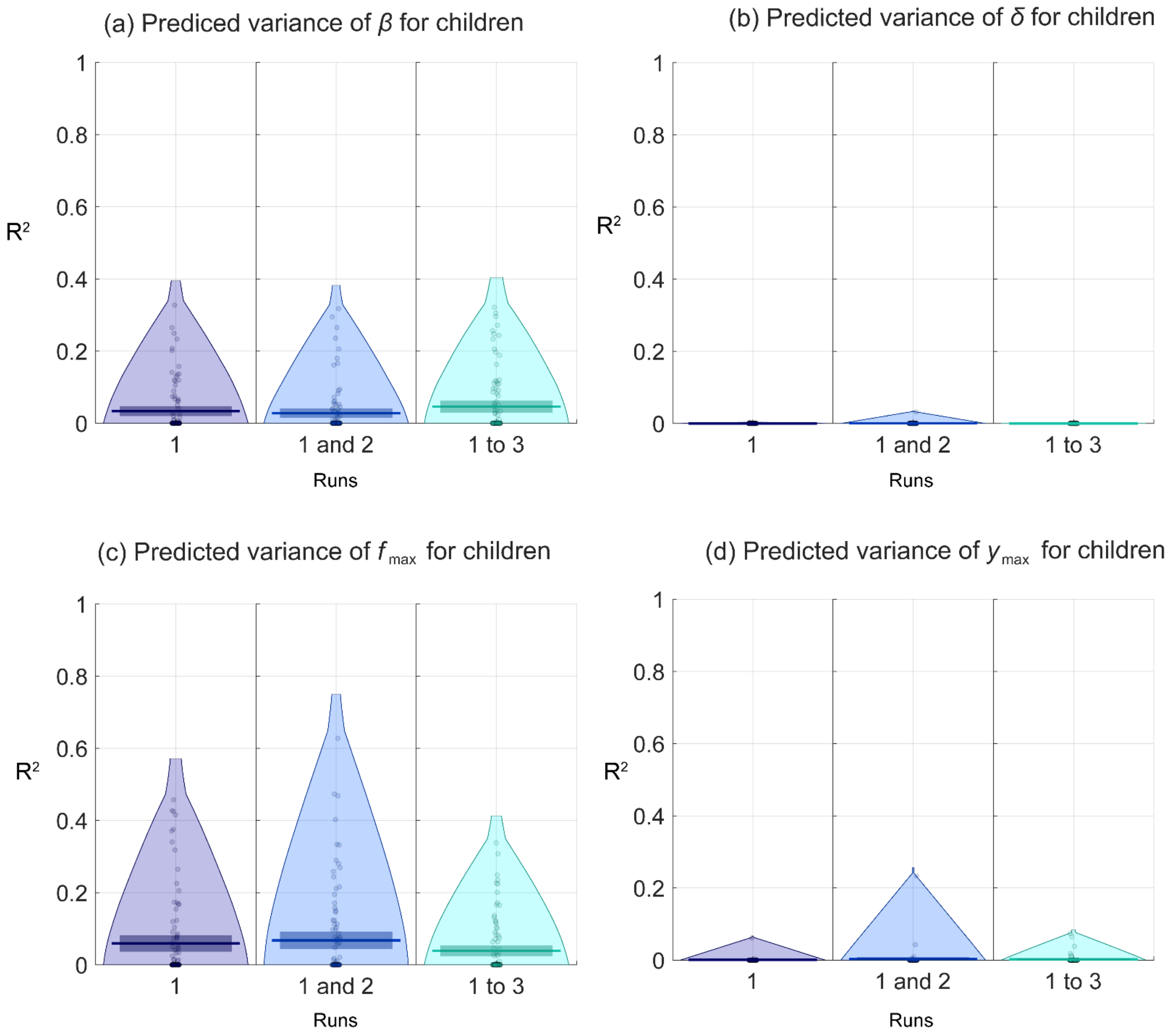

Figure S4 – The blue pirate plots show the explained variance (R^2) of the CSF parameter predictions for children participants: in (a) of β, the truncated log-parabola's full-width-at-half-maximum; in (b) of δ, the curve's truncation parameter; in (c) of f_{max}, the spatial frequency at the curve's maximal height; and in (d) of y_{max}, the curve's maximal height. The darkest, middle and lightest blue pirate plots show the R^2 of the predictions from models based, respectively, on the first, on the first and the second, and on all three three-click CSF runs. The pirate plots with warm colors show the null hypothesis obtained using a permutation test.
